# The Role of Peptide Hormones Discovered in the 21st Century in the Regulation of Adipose Tissue Functions

**DOI:** 10.3390/genes12050756

**Published:** 2021-05-17

**Authors:** Paweł A. Kołodziejski, Ewa Pruszyńska-Oszmałek, Tatiana Wojciechowicz, Maciej Sassek, Natalia Leciejewska, Mariami Jasaszwili, Maria Billert, Emilian Małek, Dawid Szczepankiewicz, Magdalena Misiewicz-Mielnik, Iwona Hertig, Leszek Nogowski, Krzysztof W. Nowak, Mathias Z. Strowski, Marek Skrzypski

**Affiliations:** 1 Department of Animal Physiology, Biochemistry and Biostructure, Poznań University of Life Sciences, 60-637 Poznan, Poland; pawel.kolodziejski@up.poznan.pl (P.A.K.); ewa.pruszynska@up.poznan.pl (E.P.-O.); tatiana.wojciechowicz@up.poznan.pl (T.W.); maciej.sassek@up.poznan.pl (M.S.); natalia.leciejewska@up.poznan.pl (N.L.); mariami.jasaszwili@up.poznan.pl (M.J.); maria.billert@up.poznan.pl (M.B.); dawid.szczepankiewicz@up.poznan.pl (D.S.); magdalena.mielnik@up.poznan.pl (M.M.-M.); iwona.hertig@up.poznan.pl (I.H.); leszek.nogowski@up.poznan.pl (L.N.); kwnowak@up.poznan.pl (K.W.N.); 2 Department of Preclinical Sciences and Infectious Diseases, Faculty of Veterinary Medicine and Animal Science, Poznań University of Life Sciences, 60-637 Poznan, Poland; emilian.malek@up.poznan.pl; 3 Department of Hepatology and Gastroenterology, Interdisciplinary Centre of Metabolism: Endocrinology, Diabetes and Metabolism, Charité-University Medicine Berlin, 13353 Berlin, Germany; mathias.strowski@charite.de; 4 Department of Internal Medicine-Gastroenterology, Park-Klinik Weissensee, 13086 Berlin, Germany

**Keywords:** adropin, apelin and elabela, irisin, kisspeptin, MOTS-c, phoenixin, spexin, neuropeptides B and W, adipocytes, fat tissue

## Abstract

Peptide hormones play a prominent role in controlling energy homeostasis and metabolism. They have been implicated in controlling appetite, the function of the gastrointestinal and cardiovascular systems, energy expenditure, and reproduction. Furthermore, there is growing evidence indicating that peptide hormones and their receptors contribute to energy homeostasis regulation by interacting with white and brown adipose tissue. In this article, we review and discuss the literature addressing the role of selected peptide hormones discovered in the 21st century (adropin, apelin, elabela, irisin, kisspeptin, MOTS-c, phoenixin, spexin, and neuropeptides B and W) in controlling white and brown adipogenesis. Furthermore, we elaborate how these hormones control adipose tissue functions in vitro and in vivo.

## 1. Introduction

Over the last several years, the interest in the metabolism of adipose tissue and its physiological role has increased significantly. This seems to be due to two main causes. The first is the discovery of leptin (the first adipokine), which changed the perception of this tissue [1,2], and the second is the fact that the number of overweight and obese people has increased dramatically in the 20th century [3]. Moreover, the development of new techniques for searching for biologically active substances based on virtual models and in silico research allowed for the discovery and description of many new biologically active substances, including proteins and peptides [4]. In this short review, we provide information on the impact of several newly discovered fpeptides on the metabolism and function of adipose tissue (white adipose tissue (WAT); and brown adipose tissue (BAT) and adipocytes.

## 2. Adropin

Adropin controls glucose and lipid metabolism [5,6]. In 2008, Kumar et al., through microarray screening, identified an adropin peptide composed of 76 amino acids and found that its secretory signal peptide sequence is encoded by residues 1–33 [6,7]. Synthetic adropin, contains residues 34–76. This isoform is biologically active [8]. This amino acid sequence is highly conserved among mammals and is identical in humans, horses, rats and mice [6]. Adropin is encoded by the energy homeostasis-associated gene (*ENHO*), which is located on chromosome 9 in humans and on chromosome 4 in mice [8,9]. An abundant *Enho* mRNA expression is detectable in many areas of the brain and in the liver [6]. Adropin mRNA expression in the liver depends upon the nutritional status [10]. For instance, hepatic *Enho* expression in mice increases during the short-term intake of a high-fat diet. However, chronic exposure to a high-fat diet leads to a reduction of hepatic *Enho* expression. On the other hand, the expression of *Enho* mRNA is also low after fasting [6]. In addition to its expression in peripheral tissues, such as the pancreas, kidney, and heart [11], adropin is also present in the circulation [11,12].

GPR19, a member of the orphan G protein-coupled receptors family, is considered as a candidate for an adropin receptor [13,14]. GPR19 is a transmembrane receptor widely distributed throughout the body, including the brain, spleen, kidney, heart, liver and lung [15]. However, it needs to be pointed out that the interaction of adropin with GPR19 has not yet been documented. Furthermore, there is evidence indicating that adropin is a brain membrane-bound protein which interacts with NB-3. This interaction contributes to the development of cerebellum, which appears to be mediated via Notch1-dependent signaling [16].

The majority of the studies showed that circulating adropin levels are negatively correlated with body mass index [17,18,19,20]. Moreover, it was found that genetically engineered *Enho*-/-mice are characterized by an increased adiposity and impaired insulin sensitivity [21]. By contrast, adropin overexpressing mice fed a high-fat diet display a delayed body weight gain, compared with wild-type animals [6]. These data suggest that adropin may be involved in controlling adipose tissue functions. Recently, we showed that the adropin precursor gene, *Enho*, and its receptor, *Gpr19*, are expressed in white and brown undifferentiated and differentiated rat preadipocytes [22,23]. Moreover, *ENHO* mRNA was detected in the adipose tissue in baboons (*Papio anubis*) [10]. However, expression of *ENHO* mRNA was significantly lower as compared to other tissues such as brain or liver. Of note, *Enho* mRNA was found in murine hormone-sensing luminal cells in mammary gland [24]. In response to low temperature (4 °C) *Enho* mRNA was up regulated [24].

Furthermore, we found that adropin stimulates white and brown preadipocyte proliferation. The stimulation of white and brown preadipocyte proliferation by adropin was mediated through ERK1/2 and AKT activation, respectively. In addition, we found that, in white rat primary preadipocytes or 3T3-L1 cells, adropin suppresses the expression of *Pparγ*, *Fabp4*, and *C*/*ebpβ* at the mRNA level and attenuates lipid accumulation [22]. Similarly, we found that adropin downregulates intracellular lipid levels and the expression of *Ucp1*, *Pparγ*, and *Pgc-1α* mRNA during the differentiation of rat brown preadipocytes [23]. Keeping in mind that the abovementioned genes are involved in white and brown adipogenesis [25], these results suggest that adropin suppresses the differentiation of white and brown preadipocytes into mature fat cells.

Unfortunately, very little is known about the role of adropin in mature adipocytes. Nevertheless, there is evidence suggesting that adropin may be involved in lipid metabolism. It was found that mice overexpressing adropin and exposed to a high-fat diet display a lower mRNA expression of genes involved in lipid synthesis, such as *Pparγ*, *Lpl*, and *Fas* [6]. By contrast, the expression of genes involved in fat oxidation (*Acrp30*, *Pgc1α* and *Cpt1a*) was not affected in mice overexpressing adropin. Little is known about the role of adropin in regulating glucose metabolism in adipose tissue. However, it is worth to note that in mice with adropin deficiency, glucose uptake was similar to that observed in wild-type animals [21]. Nevertheless, more experiments are needed to elucidate the role of adropin in controlling lipid metabolism and fat tissue formation in vivo.

**Figure 1:**
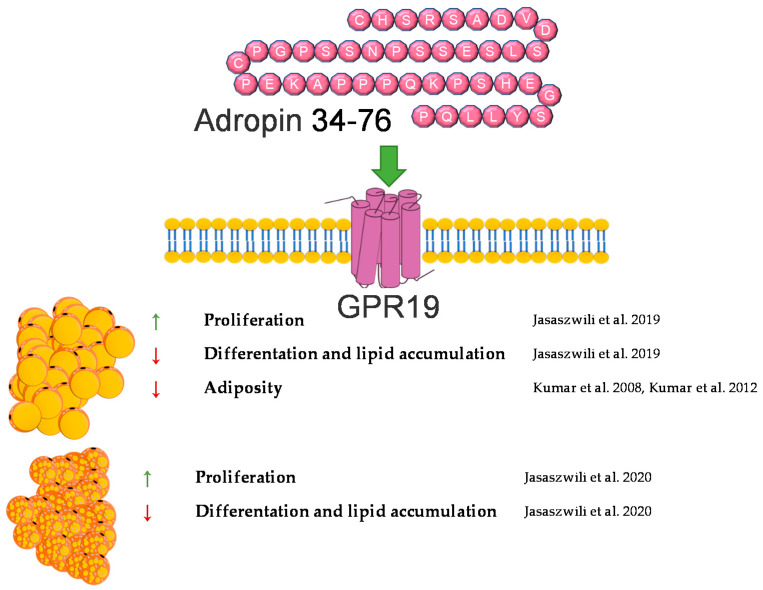
Diagram summarizing the action of adropin on WAT and BAT.

In summary, adropin deficiency may lead to adiposity. By contrast, administration or overexpression of adropin protects against body weight gain in animals fed a high-fat diet. In vitro, adropin promotes the proliferation of white and brown preadipocytes and suppresses their differentiation into mature adipocytes. A brief summary of the function of adropin on adipose tissue metabolism was provided in Figure 1.

## 3. Apelin/Elabela

APELIN (APLN) and ELABELA (ELA, Toddler, apela) are peptide hormones whose biological activity is regulated via the apelin receptor (APLNR, APJ) [26,27]. APLNR belongs to a class A (rhodopsin-like) of G protein-coupled receptors (GPCR) [26]. APLNR is widely distributed in mammals, in the central nervous system as well as in peripheral tissues [28,29]. Both agonists of APLNR—ELA and APLN—can modulate multiple intracellular signaling pathways, such as adenyl cyclase (AC) and PKA (protein kinase A) [30].

Despite the fact that these peptides are 15 years apart in terms of their discovery (APLN was discovered in 1998; ELA, in 2013), they still arouse the interest of scientists around the world [31,32].

### 3.1. Apelin

The apelin gene in the human and mouse genomes is located on chromosome X and encodes a 77 amino acid prepropeptide, pre-pro-apelin [33]. As a result of the post-translational processing of this precursor peptide, Apelin-17, Apelin-13, and [Pyr1]-Apelin-13 (with a pyroglutamate substitution at the N terminus of Apelin-13) are formed. All of these isoforms are biologically active. Previous studies showed that apelin and the APJ receptor are widely expressed in all mammalian peripheral tissues, such as the stomach, heart, lung, skeletal muscle, etc., as well as in some regions of CNS, e.g., in the brain, including the extrahypothalamic structures, such as the piriform cortex, the nucleus of the lateral olfactory tract, the central grey matter, the pars compacta of the substantia nigra, the dorsal raphe nucleus, and the entorhinal cortex [33,34,35,36,37,38].

**Figure 2:**
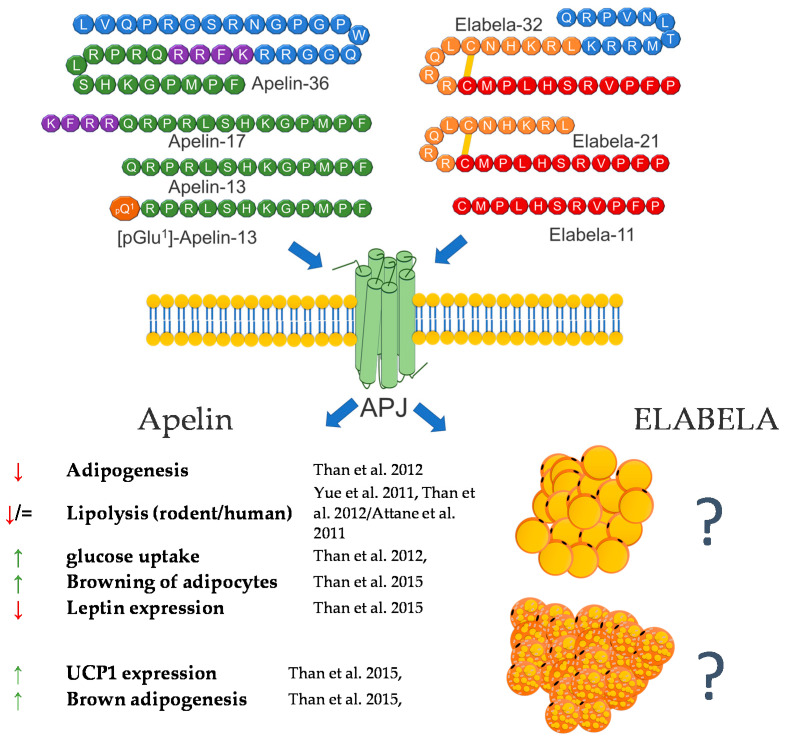
Diagram summarizing the action of apelin and elabela on WAT and BAT.

Apart from research addressing the role of apelin in regulating carbohydrate-lipid metabolism and food intake, the link between apelin and the regulation of adipocyte’s functions was reported soon (2 years) after apelin’s discovery [38,39,40,41,42]. The current knowledge regarding the role of apelin in the regulation of adipocytes is derived from in vitro and in vivo experiments. As mentioned above, in 2001, Tatemoto et al. investigated the effect of apelin on lowering blood pressure and showed that apelin is expressed in adipocytes in rats, which was also confirmed one year later by De Falco and coworkers in human fat tissue [34,38]. Since then, many functions of apelin in regulating fat cells have been described, and all of them confirm that this peptide is a potent regulator of the metabolism of this tissue. Most studies are concerned with the role of this peptide in obesity. Later research of Boucher et al. proved that apelin is upregulated by insulin and obesity and could be secreted by adipocytes [35]. After these findings, apelin has been considered as a new adipokine. Later research also showed that APLN expression is regulated by many factors, whose balance is disturbed during the occurrence of many diseases related to adipose tissue. One of them is TNF*α*. The research showed that APLN mRNA expression is increased in response to this factor in human and mouse adipose tissue [43]. Other research also showed that apelin mRNA is downregulated by glucocorticoids in 3T3-L1 adipocytes [44] and upregulated by hypoxia [45], eicosapentaenoic acid [46], inflammation (LPS) [47], and the transcriptional proliferator-activated receptor *γ* (*PPARγ*) coactivator 1*α* (*PGC-1α*) [48]. Moreover, in 2011, it was found that a blockade of the renin–angiotensin system ameliorates apelin production in 3T3-L1 adipocytes [49]. Apart from the description of the influence of many different factors on the expression of apelin, many effects of this peptide on the functioning of adipocytes were also demonstrated. One of the basic processes in adipose tissue is lipolysis and lipogenesis. In 2011, Yue et al. showed that apelin increases lipolysis in 3T3-L1 cells via the activation of AMP-activated protein kinase and the Gq and Gi pathways. In this study, the authors also showed that apelin-knockout mice have an increased abdominal adiposity and higher circulating FFA levels, and apelin infusion abolished this effect [50]. These findings were confirmed by other research groups, which demonstrated that APLN inhibits adipogenesis (through MAPK kinase/ERK dependent pathways) and basal lipolysis (through basal the AMP kinase-dependent enhancement of perilipin expression), and acute/hormone-stimulated lipolysis decreases the perilipin phosphorylation in 3T3-L1 and rat primary isolated preadipocytes. Moreover, it was found that apelin stimulates glucose uptake via the PI3K/Akt pathway, thus improving insulin resistance in 3T3-L1 adipocytes [51,52]. On the other hand, ex vivo research performed on human isolated adipocytes did not prove the stimulating role of APLN in lipolysis but showed that APLN stimulates the glucose uptake in these cells, thus increasing AMPK phosphorylation [53]. In 2015, Than and coworkers, using primary rat and mouse brown preadipocytes isolated from interscapular BAT, as well as human preadipocytes, also proved that APLN enhances brown adipogenesis and the browning of white adipocytes via the PI3K/Akt and AMPK signaling pathways [54]. A brief summary of the function of apelin in adipose tissue metabolism was provided in Figure 2.

### 3.2. Elabela/Toddler

The function of this peptide in the context of adipose tissue metabolism is still largely unknown. However, based on the literature data and its effect on metabolic pathways, such as the regulation of the SIRT3-mediated inhibition of oxidative stress through Foxo3a deacetylation [55] or the negative association between blood glucose level and ELA, these are grounds to assume that APLN and ELA may also be involved in the metabolism of adipose tissue [56,57]. In summary, the literature data indicate that both APLN and ELA are potent regulators of adipose cell metabolism. However, the effect of ELA is still unclear, and future studies are required.

## 4. Irisin

Irisin, a newly reported molecule, was firstly described in animals, and since then, it has been one of the peptides of increasing research popularity. As part of the adipomyokines class, it interacts with adipose tissue and muscle tissue. Irisin was firstly described by Bostrom in 2012 at Harvard University. During this research, irisin was shown to be a myokine that is up-regulated by exercise [58]. The precursor of irisin, the FNDC5 protein, is built of residues of 209 aa, consisting of an N-terminal signal sequence, a fibronectin-type III domain, an unidentified region, a transmembrane domain, and a C-terminal part. The release of irisin is connected to the cleavage of the extracellular part of the FNDC5 protein [59]. Later research also demonstrated that irisin is expressed and produced by human muscle cells [60]. For many years, the receptor through which irisin exhibits its biological activity remained unknown. However, in 2018 Kim et al. showed that the effects of irisin in bone and fat are mediated via *α*V integrin receptors. In addition, Kim et al. pointed out that the interaction of irisin with its receptor triggers a cascade of signaling pathways including phosphorylation of focal adhesion kinase (FAK), protein kinase B (AKT), and cyclic AMP response element-binding protein (CREB) [61]. Irisin is responsible for the process of browning of white adipose tissue. Which occurs during a physical exercise. Exercise leads to an increased expression of the FNDC5 gene, as a result of an intensified expression of peroxisome proliferator-activated receptor *γ* coactivator 1-*α*-PGC-1*α* [62,63]. Subsequently, irisin is released as a product of the proteolysis of FNDC5. This protein stimulates the process of browning of white adipocytes as an effect of activating the protein membrane of mitochondrium-uncoupling protein 1-UCP1 [64]. These results were also confirmed by Li et al. in 2019 [65]. Initially, it was shown that irisin increases the mRNA expression of UCP-1, PGC1*α*, PRDM16, TMEM26, and CD137 in human adipocytes and subcutaneous white adipose tissue. This effect was not observed in visceral/brown adipose tissue or their derived mature adipocytes [65].

In research aimed at explaining the mechanisms of action of irisin, Zhang et al. showed that the protein induces the phosphorylation of MAPKs as a regulation of the process of browning. Moreover, it was also shown that irisin exerts dual effects on the browning and adipogenesis in human adipocytes. In mature white adipocytes, irisin increases thermogenesis (this effect was mediated by p38/ERK signaling); however, in precursory cells, irisin inhibits the formation of adipocytes and promotes osteogenesis processes [65,66,67,68].

A few studies also proved that irisin is able to regulate basic adipose tissue processes, such as lipolysis, lipogenesis, and glucose uptake. Research performed on human adipocytes showed that irisin inhibited basal- and isoproterenol-stimulated lipolysis [69].

Irisin has a variety of effects on metabolism. It intensifies metabolism by increasing oxidative metabolism, resulting in an increased glycolysis over a short period (up to 4 h). However, it is interesting that a 24 h irisin treatment inversed this effect, and glycolytic metabolism was reduced [70]. Xiong et al. showed that using the cAMP–PKA–HSL pathway, the protein increases lipolysis [71]. By contrast, some research showed that irisin had no effect on lipolysis in a 3T3-L1 cell line [72]. The studies of Ma et al. reported that the protein is connected to adipocyte differentiation and can suppress adipogenesis through the regulation of the Wnt metabolic pathway [73]. Gao et al. found that irisin is responsible for the up-regulation of adipose triglyceride lipase. The same study reported the same mechanism of action for fatty acid-binding protein 4 [74]. Irisin regulates cholesterol synthesis through its inhibition of SREBP and by activating AMPK [75]. The protein is also responsible for reducing the levels of leptin and increasing ghrelin levels [76]. It was shown that irisin increases fatty acid *β*-oxidation in muscle tissue [77]. It should also be noted that in contrast to animal data, the role of irisin in the context of the regulation of human adipose tissue functions remains very controversial. The main reason for the controversies is based upon a questionable translation of the irisin gene. Analyses of genomic DNA, mRNA and expressed sequence tags revealed that FNDC5, as the gene encoding the precursor of irisin in human genome, shows a mutation in the conservative start codon ATG to ATA. Thus, the translation into the protein may be impaired. The authors suggest also that FNDC5 should be annotated as a transcribed pseudo-gene that has lost its ability to be effectively translated into the full-length protein [78].

**Figure 3:**
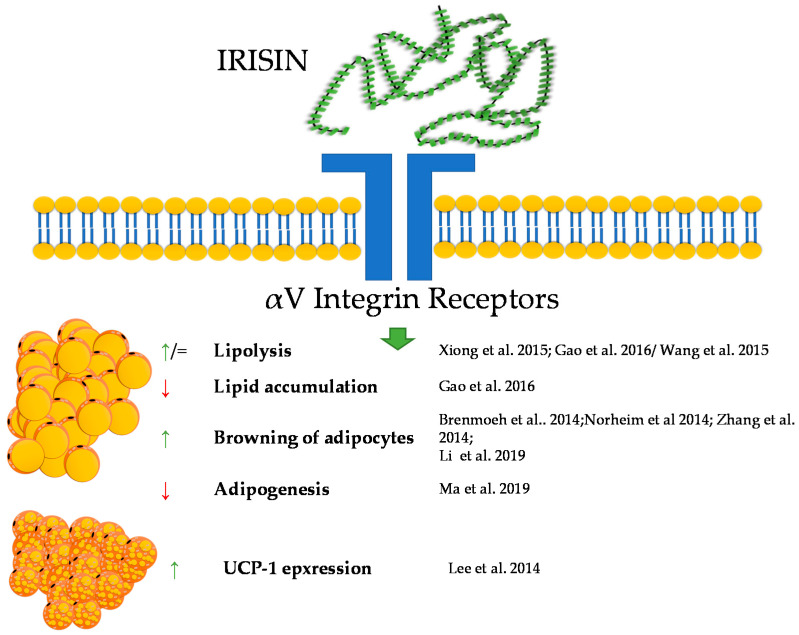
Diagram summarizing the action of irisin on WAT and BAT.

Taken together, irisin, in a similar way to the previously described peptides, shows a great potential for the regulation of the metabolism of adipose tissue; however, still, little is known about its influence on this tissue. A brief summary of the function of irisin in adipose tissue metabolism was provided in Figure 3.

## 5. Kisspeptin

Kisspeptin (KP) is encoded by the *KISS1* gene and is formed as a 154 amino acid protein precursor [79]. After cleavage, several forms of biologically active kisspeptins are created. The *KISS1* gene and its receptor, GPR54 (known as Kiss1r), were discovered in 1996 and 1999, respectively [80]. The main product of KISS1 gene is a 54 amino acid protein. However, also other cleavage peptides such as KP10, 13, and 14, bind to the GPR54 receptor with similar high affinities, thereby causing biological effects [81]. The expression of GPR54 in human tissues as well as in rodents is wide. So far, the expression of GPR54 has been described in different regions of the brain [82,83], as well as in numerous peripheral tissues such as liver, pancreas or fat [84,85]. Moreover, studies indicate that GPR54 is able to promote adipocytes differentiation and fat accumulation in mice via a MAP kinase pathway [85].

**Figure 4:**
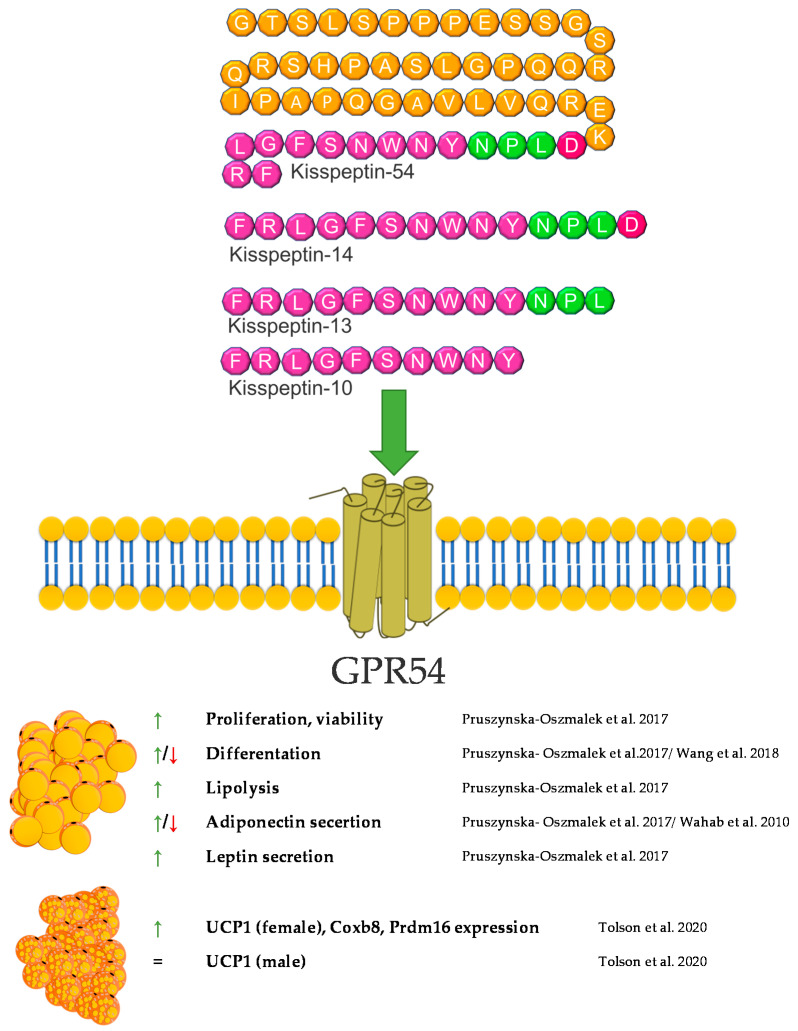
Diagram summarizing the action of kisspeptin on WAT and BAT.

Kisspeptin has been recognized as an important maturation and reproductive factor acting in the central nervous system. Its expression was also demonstrated in peripheral tissues involved in carbohydrate-lipid metabolism, i.e., in adipose tissue [86], the liver [87], and the pancreas. Kisspeptin is the link between reproduction and the body’s energy status. Reproduction is costly in terms of energy consumption, and maturation requires an adequate supply of nutrients. The lack of food and energy leads to fertility disorders and even infertility [88]. This regulation is based on many mechanisms; however, it seems that kisspeptin is one of the strongest factors [81]. The influence of Kisspeptin on energy expenditure occurs not only through a direct interaction with is target tissues but also through an effect on the food behavior. The impact on food intake involves the regulation of hypothalamic neuropeptides and neurotransmitters such as neuropeptide Y (NPY). Research performed by Orlando et al. in 2018 showed that KP10 treatment of rat hypothalamic cell line Hypo-E22 resulted in an increased NPY mRNA expression [89]. Energy metabolism is based on the main energy reservoir—adipose tissue. Research on kisspeptin in the context of adipose tissue is unclear. Both, GPR54 and kisspeptin are expressed herein [90]. Interestingly, the expression of KP is independent of the hypothalamus but may depend on the sex hormones. It can act locally on adipocytes or on the entire body [90]. In vitro studies have shown that KP10 enhances lipolysis, which leads to a lower accumulation of TGs in white adipocytes. Additionally, it also reduces glucose uptake, which confirms its action in reducing fat accumulation [86]. On the other hand, the opposite effect was demonstrated in a model of mice with a lack of the GPR54 receptor. The experiment showed that a lack of GPR54 inhibits the accumulation of TGs, and the growth of adipocytes is caused by a high-fat diet [85]. KP may also act as regulators of adipokine secretion by adipose tissue. According to the literature, KP reduces the secretion of adiponectin in 3T3-L1 cells [86], but an increase in the concentration of adiponectin was observed in/after peripheral administration in Rhesus monkey [91]. Interestingly, kisspeptin is also present in brown adipose tissue. Mice with a global kisspeptin receptor knockout have a higher body weight than mice lacking the receptor only in brown adipose tissue [92]. Other studies have shown that the effect of kisspeptin in brown adipose tissue differs between males and females [93]. Undoubtedly, this information needs further analyses to confirm the direction of kisspeptin’s action, both at the level of adipocytes and the whole organism. Another aspect of the body’s energy metabolism is the involvement of kisspeptin in food intake. Dong et al. showed that the intraperitoneal dose of KP10 reduces food intake in the first 4 h after injection. An increase in the level of leptin, resistin and insulin was also observed [94]. A similar effect in reducing food intake was obtained by Stengel et al., after administering kisspeptin centrally in mice [95]. The effect of weight loss was also observed by Sahin et al. Female rats treated with kisspeptin were characterized by a lower body weight and a lower concentration of fatty acids in the serum [96]. External factors also influence the level of kisspeptin. In male mice, obesity and high leptin levels have been shown to reduce the expression of the *GPR54* and *Kiss1* genes [97]. The decrease in kisspeptin expression in ovaries was also observed in females exposed to a high-fat diet [98]. The decrease in the expression of KP and its receptor may also be caused by metabolic disorders, such as type 1 diabetes mellitus (DM1) and type 2 diabetes mellitus (DM2) [84]. However, in humans, it has been confirmed that obesity in women is correlated with a low level of KP in the serum [99]. The involvement of KP in lipid metabolism is certain. Its action, however, is difficult to explain linearly, and due to its expression in many tissues, it can create a vicious circle in the body’s energy metabolism. More research is needed to clarify the effects kisspeptin achieves in various reproductive and metabolic disorders and how these disorders may affect the levels of kisspeptin in the central system and in peripheral tissues. A brief summary of the function of kisspeptin in adipose tissue metabolism was provided in Figure 4.

## 6. Mots-C

MOTS-c is a peptide that is still poorly understood. It was discovered in 2015 [100]. According to the available reports, it seems to be an important regulator of energy balance and the metabolism of carbohydrates and lipids. MOTS-c can have a direct effect on lipid metabolism and adipose tissue by affecting these metabolic pathways and an indirect effect by affecting carbohydrate metabolism, which results in lipid pathway changes. It is worth noting that the origin of MOTS-c, which is the 12S RNA gene sequence, is located in the mitochondrial DNA, suggesting a possible important relationship with the metabolism of each cell of the body. A first report showed an in vitro influence of MOTS-c on lipid metabolism. The fatty acid oxidation was increased via the phosphorylation-induced inactivation of acetyl-CoA carboxylase (ACC), and higher levels of carnitine shuttles and reduced levels of essential fatty acids were observed. Finally, MOTS-c prevented high-fat diet-induced obesity in mice [100]. A similar effect of increasing fatty acid oxidation was postulated due to the increase in carnitine palmitoyltransferase I (CPT1A) expression after MOTS-c treatment [101]. A subsequent report also showed anti-obesity properties of MOTS-c. [102]. Lu et al. demonstrated that MOTS-c regulates adipose tissue metabolism in ovariectomized mice. It has been showed that MOTS-c attenuates lipid accumulation in fat tissue through the AMPK pathway, decreases plasma lipid, enhances lipid catabolism, and, finally, reduces fat mass and prevents body weight gain.

**Figure 5:**
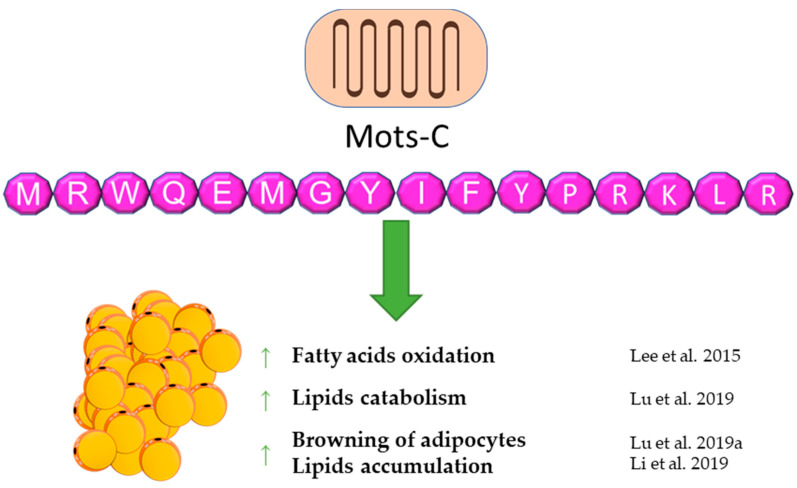
Diagram summarizing the action of Mots-C on WAT.

A novel and important finding is the activating influence of MOTS-c on brown adipose tissue [103]. Additionally, MOTS-c can stimulate the transformation of white adipocytes into brown adipocytes and activate brown adipose tissue by enhancing the expression of thermogenic genes, which leads to cold adaptation [102]. According to the previous data, a decrease of lipid accumulation in subcutaneous tissue and liver was also observed in mice treated with galactose, which is a model of aging [104]. On the other hand, the increasing effect of lipid infusion on the MOTS-c serum level was shown in human subjects. This suggests a feedback loop between MOTS-c and lipids, which may be a significant part of energetic homeostasis [105]. The abovementioned results of studies reveal an important contribution of MOTS-c to energy balance and adipose tissue functioning. A brief summary of the function of Mots-C in adipose tissue metabolism was provided in Figure 5.

## 7. Neuropeptide B and Neuropeptide W

Neuropeptide B (NPB) is a hormone peptide, originally identified as an endogenous ligand for the G protein-coupled GPR7 receptor (NPBWR1) [106,107,108]. NPB is composed of 29 amino acids with a unique modification at the C-6 position of the indole ring of the N-terminal tryptophan bromination. Neuropeptide B is proteolytically cleaved from the 125 amino acid prepro-NPB precursor, and in humans, it gives two amino acids of different lengths, NPB23 and NPB29 [107]. Nevertheless, NPB29 was only described in nonhuman mammals. Interestingly, in chickens, prepro-NPB gives a 28 amino acid mature peptide, which is different from the 23 or 29 amino acids of the NPB of mammals [109]. The biological effects of NPB are mediated through the activation of two GPCR receptors, called NPBWR1 (GPR7) and NPBWR2 (GPR8), which are highly similar to each other, sharing 59% amino acid identity, and they also bind a paralogous peptide, named neuropeptide W (NPW) [108]. NPW is also present in two isoforms of different lengths—of 23 (NPW23) and 30 (NPW30) amino acids, which are identical in the N-terminal 23 amino acid sequence. Both are produced from the prepro-NPW precursor [110] and were discovered in the same year as NPB (2002) [111]. Both the NPW23 and NPW30 isoforms were detected in human, rat, mouse, pig, and chicken species [108,109,112,113,114].

It was found, that NPB purified from bovine hypothalamus extracts, binds to and activates human NPBWR1 and NPBWR2 with median effective concentrations (EC_50_) of 0.23 nM or 15.8 nM, respectively [108]. Furthermore, synthetic human NPW binds to and activates human NPBWR1 and NPBWR2 with EC_50_ values of 0.56 nM or 0.51 nM, respectively [108]. Both receptors are members of G-protein-coupled receptor superfamily, and upon ligand binding various intracellular processes in different cell types are activated, e.g., cAMP downregulation in CHO cells [115] or stimulation of cAMP in the human adrenocortical cells [116]. Both, NPB and NPW can activate adenylate cyclase/PKA-dependent cascade, however only NPB activates the PLC/PKC-dependent cascade [117]. NPB can enhance phosphorylation of ERK1/2 MAP kinase in INS-1E cells [118] and can stimulate p-38 MAP kinase in rat brown preadipocytes [119].

Neuropeptide B is widely expressed in the organism, predominantly in the central nervous system [106,120,121], as well as in peripheral tissues, such as the gastrointestinal system, heart, ovary, testes, adrenals, pancreatic islets, and fat tissue [118,121,122,123]. Neuropeptide B was implicated in controlling appetite [108], inflammatory pain [124], stress hormone secretion [125], and the stimulation of aldosterone [126], insulin [118], progesterone, and testosterone [127].

The NPW tissue distribution is similar to that of NPB. Predominantly, NPW is expressed in CNS, especially in several nuclei of the hypothalamus [114,121,128,129]. Among the peripheries, neuropeptide W is expressed in the heart, stomach, pancreas, adrenal and thyroid glands, ovary, testes, and adipocytes [107,114,122,130,131]. Neuropeptide W is involved in the regulation of food intake and the endocrine system. The ICV chronic administration of NPW led to a reduced food intake and weight loss in rats [132]. Moreover, NPW signaling is essential in response to stressful stimuli [133]. An in vivo study showed that, in rats, NPW but not NPB decreases blood insulin and leptin concentrations [131]. Similar to NPB, NPW increased cortisol secretion in human- [133] and rat-cultured adrenocortical cells [126].

Animal studies showed that mice with depleted NPBW1 (NPBW1-/-) and neuropeptide B-deficient mice (NPB-/-) develop mild adult onset obesity [124,134]. These data clearly demonstrated that NPB/NPBW1 signaling is involved in controlling body weight possibly via the regulation of the formation/differentiation and functioning of adipocytes.

We found that rodent adipocytes express the NPBWR1 receptor [130]. Moreover, NPB and NPW mRNA are expressed in preadipocytes but not in mature white adipocytes. It was found that both neuropeptides reduce mRNA expression and the secretion of leptin in isolated mature rat adipocytes and promote lipolysis. In addition, it was found that only NPB stimulates the mRNA expression and secretion of resistin. The involvement of neuropeptide B in resistin secretion suggests that NPB may contribute to the modulation of pathophysiological processes dependent on resistin, such as insulin resistance, diabetes, atherosclerosis and cardiovascular diseases, autoimmune disease, asthma, and non-alcoholic fatty liver disease [135]. Moreover, since both peptides may inhibit food intake and leptin secretion, it cannot be excluded that these peptides may be involved in controlling leptin-mediated food intake and energy homeostasis [130,136].

Recently, we assessed the role of neuropeptide B in the functioning of rat brown adipocytes [119]. Neuropeptide B and NPBWR1 were detected at the mRNA and protein level in brown preadipocytes isolated from rats. Moreover, we found that neuropeptide B increases the viability and proliferation of cultured brown preadipocytes. In addition, it was demonstrated that NPB stimulated the expression of *Prdm16* and *Ucp1*, but it suppressed the mRNA expression of antiadipogenic preadipocyte factor 1 (*Pref1*), indicating that this peptide promotes the differentiation of rat brown preadipocytes differentiation into mature brown adipocytes. The stimulation of the Ucp1 mRNA expression was mediated through p38 kinase activation.

**Figure 6:**
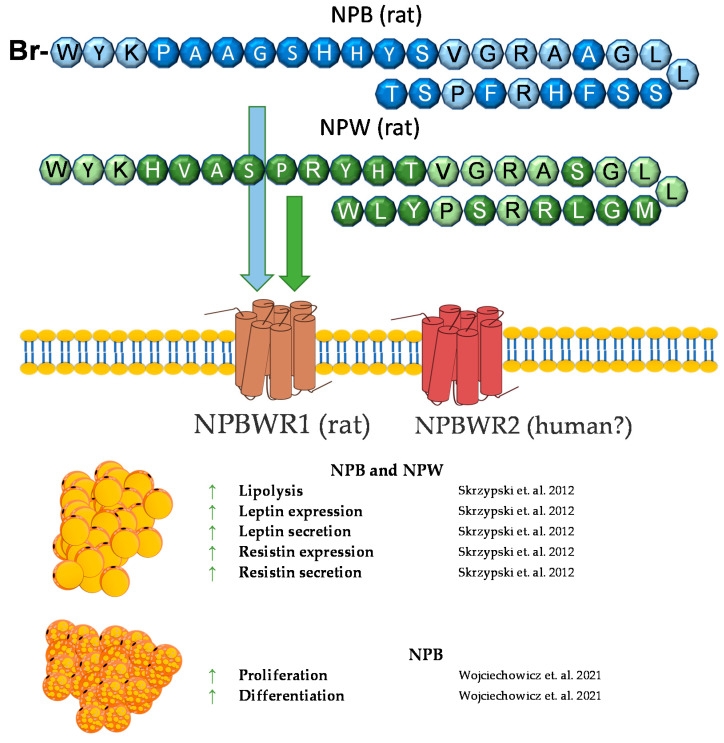
Diagram summarizing the action of NPW and NPB in WAT and BAT.

In summary, NPB and NPW exert direct effects on adipocytes. In vitro studies demonstrated that NPB and NPW promote lipolysis and suppress leptin expression and secretion. Moreover, NPB promotes brown adipogenesis in vitro. By contrast, little is known about the effect of the NPB/NPW system on adipose tissue in vivo. A brief summary of the function of NPB and NPW in adipose tissue metabolism was provided in Figure 6.

## 8. Phoenixin

Phoenixin (PNX) is a neuropeptide cleaved from the small integral membrane protein 20 (SMIM20). It was identified in 2013 by G. Yosten et al. based on the bioinformatic approach, and the data are collected in the Human Genome Report [137]. The two main PNX isoforms are the 14 and 20 long amino acids. The PNX peptide sequence is highly interspecies conservative. The PNX14 amino acid sequence is the same in humans, bovines, pigs, mice, rats, and chickens, while the sequence of PNX20 in humans and rodents differs in one amino acid. Based on spectrometric mass analysis, PNX20 expression is predominant in the hypothalamus, and PNX-14 predominates in the heart and spinal cord [137,138]. Despite the observed differences in expression depending on the tissue, it is assumed that both PNX isoforms have a very similar biological activity [139,140]. The putative receptor of PNX is G-protein-coupled receptor 173 (GPR173) [141], belonging to the Super Conserved Receptor Expressed in the Brain (SREB) family, also known as SREB3. It has been selected by the “deductive ligand-receptor matching strategy” developed by Yosten’s research team [142]. Although GPR173 is termed as PNX receptor in many research data, there is no definitive evidence of PNX-selective binding to this receptor, nor is it known whether it is the only receptor through which PNX exerts biological actions.

PNX is highly involved in the reproduction system, where it modulates GnRH and LH secretion and regulates the duration of the diestrus stage in rats [137,141,143]. Moreover, it stimulates the maturation of ovarian follicles and increases the number of ovulated oocytes [144]. The PNX serum level positively correlates with LH, FSH, and progesterone in women with polycystic ovarian syndrome (PCOS) [145]. A higher PNX concentration in serum was observed in obese men [146]. Moreover, a positive correlation of the PNX level and BMI was shown in women with PCOS [145]. Based on this information, our group investigated the effects of PNX on white adipogenesis in vitro [147]. We showed the mRNA expression of the PNX precursor peptide, Smim20, and Gpr173 in the 3T3-L1 cell line and in rat primary preadipocytes, as well as the PNX production and secretion from 3T3-L1 and rat primary adipocytes. Furthermore, PNX increased the proliferation and differentiation of preadipocytes and decreased cell death. The PNX preadipocyte differentiation stimulation mechanism was dependent on cAMP/Epac signaling [147]. The detection of PNX and Gpr173 at the mRNA and protein levels was also shown in periovarian adipose tissue in rats [148]. Due to the high metabolic importance of adipose tissue in PCOS [149] and the increased level of PNX in PCOS women [145], the presence of PNX in a letrozole-induced PCOS rat model was investigated [148]. An increased mRNA expression of Smim20 and decreased expression of Gpr173 in periovarian adipose tissue in a PCOS rat model [148].

**Figure 7:**
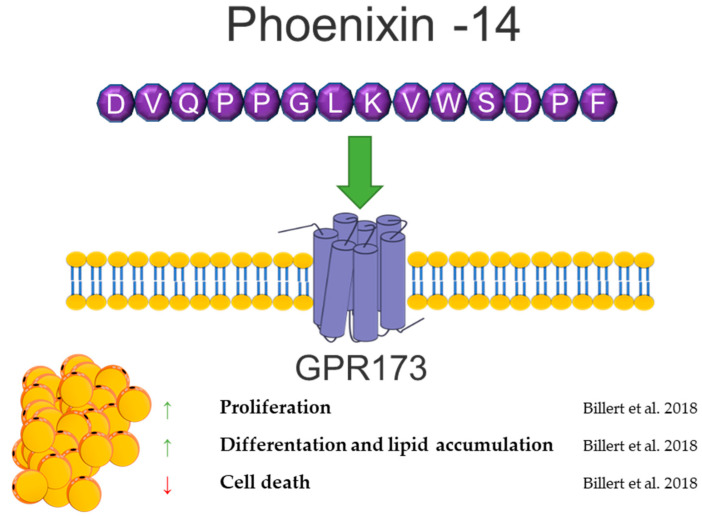
Diagram summarizing the action of phoenixin in WAT.

Based on data showing a positive correlation of the PNX serum level with BMI and stimulation of white adipogenesis in vitro, the contribution of this neuropeptide in controlling adipose tissue physiology cannot be excluded. Furthermore, in vitro and in vivo studies are needed to determine whether the PNX involved in energy metabolism is physiologically relevant. A brief summary of the function of PNX in adipose tissue metabolism was provided in Figure 7.

## 9. Spexin

Spexin (SPX) is a novel 14 amino acid peptide that was first identified in 2007 in the human proteome using bioinformatics based on hidden Markov model screening [150]. Subsequent studies on this peptide showed that it is an agonist of two isoforms of the galanin receptor. SPX activates isoform 2 (GALR2) and isoform 3 (GALR3) of the galanin receptor, whereas the isoform 1 (GALR1) activation of this receptor by SPX has not been demonstrated [151].

Despite the fact that the research on this peptide is relatively recent, the knowledge about it has systematically increased. Research conducted on many different groups of animals showed a wide expression of this peptide in many different tissues, including fat tissue/adipocytes [152,153,154,155,156,157,158]. The first study that connected SPX with fat tissue metabolism were experiments performed by Walewski et al. in 2014. They showed that the SPX gene is one of the most downregulated genes in adipose tissue during obesity and that SPX administration reduced food intake in mice [159]. Other research has also shown that the SPX concentration in serum is downregulated during obesity and diabetes [99,160,161,162]. In isolated adipocytes and the 3T3-L1 cells SPX had no effect on the proliferation or cell viability; however, it was able to regulate adipogenesis, lipolysis, lipogenesis and glucose uptake [154,159,163].

Walewski et al. also showed that SPX reduces the uptake of long-chain fatty acids by adipocytes [159]. Our research from 2018 confirmed this research and also proved that SPX inhibits adipogenesis through the downregulation of the expression of proadipogenic genes, such as *Pparγ*, *C*/*ebpα*, *C*/*ebpβ*, and *Fabp4*, in 3T3-L1 cells, stimulates lipolysis, increasing hormone-sensitive lipase (HSL) phosphorylation, and inhibits glucose uptake and lipogenesis in 3T3-L1 and isolated human and rat [154,163]. The effect on fatty acid uptake was also confirmed in hepatocytes [164]. Another important study on the effect of SPX on the metabolism of adipose tissue is the study by Gambaro et al. In their research, SPX treatment of mice reduced adipocyte hypertrophy and M1 macrophages and subtypes (M1a and M1b), which caused an improvement of adipose tissue inflammation by affecting proinflammatory markers: the mRNA expression of IL-6, IL-1*β*, and TNF-*α*. Moreover, in vitro research by the same laboratory demonstrated that the reduction of the activation of adipose tissue macrophages (ATMs) is mediated directly and through cross-talk between adipocytes [165]. This same effect was noted in serum in rats [166]. Our research performed in broiler chickens also proved that SPX expression is regulated by fasting time in fat tissue, the liver, and muscle, which also indicates that the SPX system could be a potent regulator of carbohydrate and lipid metabolism in birds [153].

**Figure 8:**
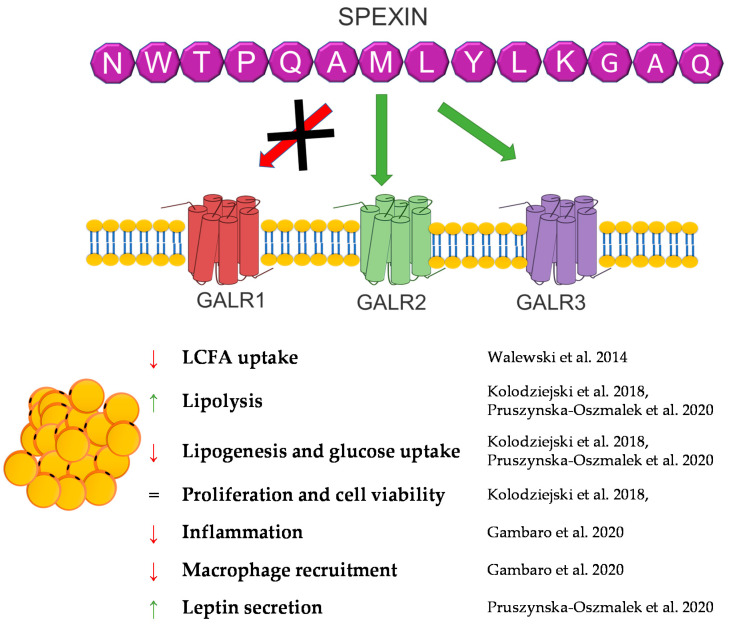
Diagram summarizing the action of SPX in WAT.

In summary, SPX is a strong regulator of fat tissue metabolism, and a deficiency of this peptide leads to obesity. However, it is interesting that SPX causes a decrease in body weight and an improvement in lipid metabolism in vivo. Still, nothing is known about the role of SPX in brown adipose tissue metabolism, leaving plenty of room for new discoveries concerning the physiological role of SPX. A brief summary of the function of SPX in adipose tissue metabolism was provided in Figure 8.

## 10. Concluding Remarks

Despite the fact that the functioning of adipose tissue has been researched for many decades, it remains a mystery. This is because, from year to year, new biologically active substances produced by the body are discovered that can affect the functioning of this tissue, and the knowledge about those already discovered is still insufficient. In this review, we tried to briefly present the current knowledge on the role of selected peptides (adropin, apelin, elabela, irisin, kisspeptin, MOTS-c, phoenixin, spexin, and neuropeptides B and W) in the metabolism of adipose tissue cells. We hope that this will allow for the systematization of the knowledge on these peptides/proteins and help to establish new lines of research on their role in the metabolism of fat tissue. These studies are particularly important in the context of the increasing number of overweight and obese people, as well as the complications associated with these conditions.

## Data Availability

Not applicable.

## Abbreviations

**3T3-L1:** mouse embryonic fibroblast cell line; **AA:** Amino acids; **ACC:** acetyl-CoA carboxylase; **ACRP30:** adipocyte complement-related protein of 30 kDa; **AKT:** protein kinase B (PKB); **AMPK:** 5′AMP-activated protein kinase; **APLN:** Apelin; **APLNR, APJ:** apelin receptor; **ATMs:** adipose tissue macrophages; **BAT:** brown adipose tissue; **BMI:** cody mass index; **cAMP:** cyclic adenosine monophosphate; **C/ebp*α*:** CCAAT Enhancer Binding Protein *α*; **C/ebp*β*:** CCAAT Enhancer Binding Protein *β*; **CNS:** central nervous system; **CPT1A:** carnitine palmitoyltransferase I; **CREB:** cyclic AMP (cAMP) response element-binding protein; **DM1:** Type 1 diabetes mellitus; **DM2:** Type 2 diabetes mellitus; **ELA:** Elabela and Toddler; **ENHO:** energy homeostasis-associated gene, gene-encoding adropin; **ERK:** extracellular signal-regulated kinases; **Fabp4:** Fatty acid-binding protein 4; **FAK:** focal adhesion kinase; **FFA:** Free fatty acids; **FNDC5:** Fibronectin type III domain-containing protein 5, the precursor of irisin; **FSH:** Follicle-stimulating hormone; **GALR1:** galanin receptor isoform 1; **GALR2:** galanin receptor isoform 2; **GALR3:** galanin receptor isoform 3; **GPR173:** G-protein coupled receptor 173, Phoenixin receptor; **GPR19:** G Protein-Coupled Receptor 19, adropin receptor; **GPR54:** KiSS1-derived peptide receptor, Kisspeptin receptor, G protein-coupled receptor 54; **GPR7/NPBWR1:** G protein-coupled receptor 7, NPB receptor; **IL-1*β*:** Interleukin 1 *β*; **IL-6:** Interleukin 6; ***KISS1:*** gene-encoding kisspeptin; **KP:** Kisspeptin; **KP10:** Kisspeptin isoform consisting of 10 aa; **KP13:** Kisspeptin isoform consisting of 13 aa; **KP14:** Kisspeptin isoform consisting of 14 aa; **KP54:** Kisspeptin isoform consisting of 54 aa; **LCFA:** long-chain fatty acids; **LH:** Luteinizing hormone; **MAPK:** mitogen-activated protein kinases; **NPB:** Neuropeptide B; **NPB23:** Neuropeptide B isoform consisting of 23 aa; **NPB29:** Neuropeptide B isoform consisting of 29 aa; **NPW:** Neuropeptide W; NPW23: Neuropeptide W isoform consisting of 23 aa; **NPW30**: Neuropeptide W isoform consisting of 30 aa; **PCOS:** polycystic ovary syndrome; ***PGC-1α:*** *PPARγ* coactivator 1*α*; **PI3K/Akt:** PI3K/Akt signaling pathways; **PI3K:** Phosphoinositide 3-kinase; **PNX:** Phoenixin; **PNX14:** Phoenixin isoform consisting of 14 aa; **PNX20:** Phoenixin isoform consisting of 20 aa; ***PPARγ:*** proliferator-activated receptor *γ*; **PRDM16:** PR/SET Domain 16; **PREF1**: antidipogenic preadipocyte factor; **SIRT3:** Sirtuin 3; **SMIM20:** small integral membrane protein 20; **SPX:** Spexin; **SREB:** Super Conserved Receptor Expressed in the Brain; **TNF-*α*:** Tumor necrosis factor *α*; **UCP1:** uncoupling protein 1, thermogenin; **WAT:** White adipose tissue.
